# Extirpation of the Dental Pulp and Root Filling

**Published:** 1880-03

**Authors:** L. C. Ingersoll

**Affiliations:** Keokuk, Iowa


### THE DENTAL REGISTER.

VOL. XXXIV.] MARCH, 1880. [No. 3.

### EXTIRPATION OF THE DENTAL PULP, AND ROOT-FILLING.

BY L. C. INGERSOLL, KEOKUK, IOWA.

The history of dental science shows a gradual unfolding of the nature and functions of the dental pulp. Fifty years ago it was little known, and little appreciated. Whenever it was exposed so as to be the cause of pain, and when it was known that the death of the pulp would be followed by immediate relief, to devitalize the pulp in all conditions and degrees of exposure was the dictate of the best wisdom and the soundest practice of the times.

But when microscopy revealed the ramifications of the *vital* tissue throughout the hard structure of the teeth, and its cell-workings in the production of dentine, its functions began to be more highly appreciated, and its persistent workings were declared a necessity to the integrity of the tooth structure; and with equal confidence, its extirpation was declared a mockery of science, and a crime against nature.

The progress from nothing-worth to great, and to greatest-worth created such enthusiasm for its preservation as against extirpation, that therapeutics outran pathology, and arrived at the goal of success with a surprisingly long train of the most doubtful cases reported successfully cured.

According as we understand the structure and functions of the pulp, and the laws that control its workings, and understand the firm and enduring nature of the hard structure which is the product of its workings, will be our treatment of it in all cases when treatment is needed.

If, from any mistaken view of the importance of the pulp we have sought to save it alive in the moment of its impending death, it can scarcely be called erring on the right side, if we consider the supervening results. It might be better in certain cases to cast out the Jonah of a living pulp to save in best conditions what remains.

A few years ago, in my study of the formative organ of tooth tissue, I arrived at the conclusion that the dental pulp is variable in its functions, and wholly peculiar in its workings, and that the hard structure which it builds, usually regarded peculiar, *is* peculiar in a more important sense than is usually considered. I came to the conclusion that the pulp is not at all periods in the life of an individual of equal importance as related to the other tooth tissues, and that the argument for the preservation of the pulp, based on its necessity as an organ of nutrition, is only applicable to the earlier periods of life, and that, if it ever has a nutritive function as regards the hard tissue, it wholly ceases in after-life throughout the larger area of the tooth structure—that the generally received opinion that the hard tissue of the teeth is amenable to the law of waste and replacement of its elements is based wholly on analogy, and is without other foundation. On the other hand, I consider the fact that it is *not* subject to the general law of organic structures in regard to waste and nutrition constitutes the *chief peculiarity* of fully formed dentine.

I therefore, came to the conclusion that the loss of the pulp is not, in all cases, so damaging as the profession are wont to believe; and that there are numerous cases in which it is far better for the permanency of the remaining tooth structure to extripate the pulp at once than to attempt by long-continued doubtful means to save it alive.

I shall, therefore, *assume*, in the presentation of this paper, that there are cases where extirpation is indicated, and it will be my purpose to point out the best method, then to explain my method of filling the more difficult class of root canals thus made vacant.

Extirpation of the pulp, the first branch of my subject, has not, I am confident, gained sufficiently, the attention of the dental profession. The reason may possibly be found in the disfavor with which the profession have regarded the operation. Those who have occasionally found in their practice, cases, which in their view, demanded extirpation, have been cautious of mentioning the fact, lest they should be thought not to have caught the spirit of the age, and to be so far behind the progressionists that they might lose caste with the more advanced of the profession. Hence, that improvement which may be found in a comparison of ideas and modes of practice has not been apparent as regards this subject. Since the high-sounding notes, peal upon peal, of progress in the direction of pulp preservation have been rung in all true professional ears, this subject has seemed to many, scarcely worthy of attention. The result is that old ways and methods, which are bad ways and methods, have been practiced in comparative secresy, greatly to the detriment of dental practice, and damaging to those who have been the subjects.

When devitalization of the pulp was a more acceptable practice than it has been in later years, the term, *devitalization* seemed comprehensive of nearly, or quite, the whole operation; and all discussion of methods began, progressed and ended with the *agents* employed and the *time* occupied to effect devitalization.

We have listened long to the discussion of—How little or how much arsenic is needed? How little or how much time is required for its action? How to render the pulp insensible to its irritating and painful effects? and to a comparison of the virtues of cobalt and arsenic; and in all the discussions the *method of devitalization* was the question of chief concern.

It is quite surprising to us now that so many teeth were so kindly cared for by nature after devitalization to protect them from the usual result of alveolar abscess. While the profession to-day are far more enlightened concerning the ætiology and pathology of dental diseases and the requirements in any case, the old term *devitalization* is still much in use, and should be abandoned as too suggestive of the very inadequate methods and false ideas of former times, with respect to a very important and difficult operation in dental surgery.

The word which I have employed as the title of this branch of my subject gives us the full rounded idea of what is to be accomplished, viz.: *extirpation*; which means to root out, to eradicate, to wholly get rid of, to expel.

With this idea in mind, the work of the dentist is scarcely begun when devitalization of the pulp is accomplished; for he is required to remove every vestige of the tissue from the pulp chamber and from tbe root canals. Devitalization is a simple process, which few, if any, have difficulty in effecting, except in a few morbid cases. But extirpation is quite another operation, and in many of the teeth, is attended with very great difficulty; so great, that it is undoubtedly true that the number of operators who accomplish it with certainty in the molar teeth and in many of the bicuspids, is very small. Yet, with our present knowledge of the requirements of the case, successful treatment depends upon the *thoroughness* of the extirpation of the pulp. It is not the place for guess work and blind uncertainty; for safety depends on the certainty and completeness of the removal of the pulp tissue. How shall this be accomplished?

The general practice of the profession is sufficient warrant for the use of arsenious acid in the first step in the process, viz.: devitalization. But all should be aware that this is an all-powerful agent, and should be used with the utmost caution to prevent contact with other vital tissues than the pulp. I think nothing in the history of its use can indicate the exact quantity required to effect devitalization of a living pulp. But we do know this, that a quantity very minute has, in multitudes of cases, accomplished the purpose.

When the arsenic is applied, the cavity should be carefully sealed up so as to prevent any possible escape and action upon surrounding tissues. As a cover, I use a thick solution of gum sandarach or gum shellac on a piece of cotton. From six to twenty-four hours is sufficient time in most cases to effect devitalization—the length of time depending on the extent of the exposure and the completeness of the contact of the paste with the pulp tissue. If the cavity of decay is in the posterior wall of a molar near the gum, rather than run the risk, which is very great, of damaging the root membrane by applying the arsenic in such a locality, I seal up the cavity with gum shellac and drill a new opening through the grinding face of the tooth. Too much caution can not be used in preserving from injury the peridentium and the parietes of the alveolus, on which the future life and usefulness of the tooth must depend.

After devitalization, the next step is to secure direct and easy access to the root canals. Neglect in this particular, is always unwise. In the anterior teeth it is best, in a large number of cases, to drill in through the palatine bevel of the crown. If the decay is in the grinding surface of a molar, the opening should be enlarged with a corundum point in the engine, or by other means, to such size as will permit a full view of the entrance to the root canals. In case the cavity of decay opens posteriorly in a molar, the engine should be used to cut through the posterior angle of the crown and far enough anteriorly across the grinding surface (certainly as far as the centre), for the special purpose of getting an entrance by direct line into the roots. Thus the operator can *see* as well as *feel* his way, and know better the difficulties of his undertaking.

The next step is the removal of the devitalized tissue. In the straight round roots of the anterior teeth, this operation is so common, and attended with so little difficulty, after direct access has been gained, that it is not necessary to suggest any special modes of operating in the roots of these teeth. I am well aware that many dentists confine their root operations to this class of teeth, and therefore they never encounter the difficulties which it has been my study to overcome. The molars are the most important teeth in the mouth, and due consideration of their importance demands that especial attention be given to their preservation.

Let us surround our case with difficulties—a case taken from among the worst. Suppose it to be a second superior molar in the mouth of a patient twenty-five years of age, with firm and healthy dental organization, the cavity opening posteriorly and near the gum, the third molar in position. This case presents a complication of difficulties.

In a case with such surroundings, it may be necessary, in gaining direct access to the pulp chamber, to cut across the crown beyond the central sulcus, even to the anterior angle.

Bearing in mind now that the operation contemplated is a complete and thorough removal of the contents of the pulp chamber and of the three roots, I crave your attention while you follow me step by step.

It is not difficult to empty the pulp chamber and the palatine root of their contents; we will pass, therefore, to the buccal roots. It is quite impossible, with ordinary instruments, to enter these roots, except for a very short distance. In very rare instances, and in the mouth of young persons, the posterior buccal root may be entered far enough to lay hold of, and remove bodily, the entire nerve tissue. But this is so rare that it need not be anticipated at all in the mouth of an adult. The anterior buccal root is *still more* difficult of entrance, and to operate successfully in this root is to be in possession of sufficient skill to ensure a satisfactory operation for entire pulp removal.

I have found the barbed nerve broaches wholly impracticable for removing the tissue from the extreme portions of the root, the barbs acting as a hindrance rather than a help. The drills manufactured for the purpose of enlarging the root canals, are so large or so wanting in flexibility, as to cut through into the alveolus of a very flat root, or so badly-tempered as to be quite certain to break off in the root. In this complication of difficulties, some have resorted to chemical agents to put the fragments of nerve tissue remaining in the root in solution. Pepsin, potash and soda have been resorted to, but with very unsatisfactory results.

After removing the arsenical paste, my practice is to wash the cavity with dialyzed iron for the purpose of neutralizing the effect of any remaining portions of arsenic, which, being otherwise washed out, might come in contact with the root membrane at the neck of the tooth, and be a source of great danger to the future health of the tooth. The dialyzed iron unites chemically with the arsenic and renders it harmless. To preserve the root membrane from harm should be a matter of as much concern as the complete extirpation of the pulp.

After application of the iron, I remove the soft tissue from the pulp chamber and all from the roots that can be readily reached. I then dismiss my patient, without applying creosote or other antiseptic which might prevent decomposition, leaving only loose dry cotton in the cavity. It must be remembered that after devitalization there will be a sloughing and decomposition of all remaining fragments left in contact with the vital portion at the apex of the root, and a healing of the living portion at the point of sloughing off of the dead. These processes require time and the briefest time possible is the best. To secure this rapid sloughing and decomposition, I instruct my patient to replace in the cavity, twice a day, fresh cotton, washing out also with warm water at each removal of the cotton. This treatment should last from three days to a week. Then I give myself ample time to explore the roots. But it seems to me, vain, to direct one to do this without describing the instruments with which it is to be done. The profession have had too much advice as to *what ought* to be done, without making apparent any possible *method of doing* what is most instructively advised.

Any one who has attempted to remove wholly and thoroughly all the soft tissue from the flattened and bent roots of the superior molars, knows how inadequate are most of the instruments at hand for accomplishing it. The difficulties are so great that many a conscientious operator gives up in dispair, hoping that kind Nature will relieve him from the mortification of witnessing future unfavorable results.

For nerve and root instruments generally, I purchase jeweler's broaches. These are cheap, and with a little ingenuity can be made to serve every needed purpose. They are hexagonal in form, and from the size of a hair to any larger size that may be required. They are too hard and brittle for safe use without drawing the temper. To do this without injuring the quality of the steel, requires a very careful operation. Before heating, the instrument should be coated with soap or glutinous oil, to protect it from rapid oxidation while heating.

Where one has a hot stove in the room—not red hot—a stiff spring temper may be obtained by placing delicate hair broaches on top of the stove, then very soon grasping them with the tweezers and lifting them up *very slowly* through the hot air to the cool air above. Boiling in tallow will also give an excellent spring temper. To reduce the temper to perfect softness, and render the instrument very flexible and tough, do not thrust it into the flame of a spirit lamp. You are quite certain to fail in what you attempt in this way, and quite as certain to spoil the steel.

A better method is to coat the instruments, as before directed, then take a shovel full of glowing embers from the stove, and thrust the instruments well into the embers, to remain there until cold. For the formation of a hook on the point of a delicate instrument, I take a spring tempered broach, warm it and rub it on a bar of soap, or, to avoid the danger of over-heating, moisten the soap and rub it on the instrument; then heat an old bench file or small bar of iron to a red heat, and lay the end of the broach on it, to remain till the iron bar has cooled.

To bend the hook, bevel off the handle of a separating file, not quite to a cutting edge, place the broach against the flat side of the file handle, and holding it there firmly with pliers, bend the point of the broach down against the bevel.

A flat instrument is needed. This I make by filing into the flat form a spring-tempered broach of such size as I deem suitable; it may be very thin, while its width will give the requisite stiffness, and it will enter most of the flattened roots. With these instruments I begin my work of emptying the root canals.

First, take a straight hair-like spring-tempered broach, and pass it up to the apex, rotating if necessary to enlarge it, to learn if there remains yet any living portion of the nerve tissue, remembering that all sensation of pain is not evidence of vital tissue in the root canal. If the devitalized tissue has not yet sloughed, touching it with the instrument will cause pain at the moment of contact, which will cease when the instrument is withdrawn. If a living portion of nerve tissue remains in the canal, the pain will be likely to continue. But I do not deem it advisable again to apply the arsenious acid. I ream out the passage into the root with small, then larger broaches, until a small hooked broach will enter ; then I employ a local anæsthetic compounded of equal parts of chloroform, tincture of aconite-root and alcohol, to obtund sensibility of the tissue, when it can in most cases, be removed without serious pain. If I can not lay hold of it with the hook and remove it wholly at once, I, with the same, or with another instrument, break up the tissue and thus destroy its vitality, and after removing such fragments as I can, I leave the remaining particles for several days, to decompose, cleansing the cavity from time to time with water and wiping out with fresh cotton. This operation must not be hastened. *Time* is needed to secure a total solution of dead tissue. When the tissue is broken up mechanically we can never be *sure* of removing all by simple mechanical means. Hence, I insist most strenuously, on giving time for chemical decomposition as the only certain mode of wholly ridding the pulp chamber of every vestige of organic substance. The certainty of success depends upon this *thoroughness.*

Let us now consider the operation of filling. Any root that will permit the passage of a broach of any size through the canal to the apex, is in condition to fill, needing only to enlarge the entrance to the root, so as not to consume too much time in repeated efforts to find it. The broach should pass readily in and out.

The difficulty of the extirpation of the pulp is only second to the difficulty of filling the roots in the molar teeth. All agree that certainty of success can only be gained by filling the root canals *thoroughly* with some impervious and insoluble material ; and the absolute necessity in the case is that the canal shall be thoroughly closed at the apex. It is not ordinarily difficult to fill that half nearest the pulp chamber. But this is of comparatively little importance when the apical end of the canal is thoroughly filled. It is, therefore, evident, that any method of filling which does not with certainty reach the apex, is defective, and that which offers the fairest certainty of doing this is the best.

Will the filling materials in general use, with the *method* of using them, accomplish the purpose in the class of roots we are now considering?

While gold and tin may be successfully manipulated in the round straight roots and in some of the bicuspids, I consider it an impossibility to fill with these metals, the flattened and curved roots of the molars.

Oxy-chloride has been very favorably considered as a filling material for roots of all classes. This, too, may serve well enough in roots which can be readily entered and rapidly filled. But to have it of such consistency as to render it, when hardened, insoluble and impervious; it hardens too quickly for an operation where time is one of the elements of success, and besides, it has such an affinity, for the tooth bone, that the canal is liable to be clogged a short distance from the entrance before the more remote portion of the canal is filled.

A solution of gutta-percha in chloroform has been used with undoubted success in many cases. I consider it objectionable in two very important particulars. By its tenacious consistency it will stretch across the mouth of the canal, and the enclosed air will prevent its entrance into the roots. I do not consider experiments with it, in filling roots of teeth out of the mouth, reliable tests of its adaptability. In such cases, the apical foramen is open to permit the confined air to escape and give place to the chloroform solution. If an effort be made to expel it by force, the rapid evaporation of the chloroform leaves you a stiff, sticky material to manipulate, which is speedily beyond the operator's control. There is also the same difficulty in expelling the air when oxy-chloride is used. *Any* fluid holding in solution, solid material of any kind, and of such consistency as to form an impervious filling, will stretch itself across the narrow openings to molar roots and prevent the escape of confined air, and consequently the entrance of the fluid into the roots. Herelet me say, that of impermeable materials I consider that it matters not what is used. The only question in relation to each regards the particability of its use in making the operation of root filling a certainty in the difficult class of roots we are now considering.

I have but one other preparation to name, and that is an alcoholic solution of such gums as shellac, mastic, or copal. This solution is not new for the purpose of root filling. But the *ordinary method of using it* is open to the same objection as that already made against oxy-chloride and the gutta-percha solution. My early experience with it was attended with the same unsatisfactory results as with other materials. My method of manipulating the gum for a few years past, has brought me to the most satisfactory results that I have ever obtained in filling molar roots.

Having gained visual as well as mechanical access to the root in the manner already described, I prepare my gum in a creamy consistency, adding a small quantity of that strong antiseptic, salicylic acid, which readily dissolves in alcohol.

I select a broach of suitable size to pass to the end of the root freely, it matters not how small, and, clipping off the sharp end and rendering it smooth, I dip it into the solution and pass it carefully up the root to the end. This I do again and again. Every time the instrument passes up, it expels a portion of contained air, and leaves also a portion of gum, which was adhering to it. However minute that portion of gum may be, each successive plunge of the instrument adds to it, expelling at the same time, the air to give place to it. A pool of the gum solution may be formed in the pulp chamber, not with the anticipation that capillary attraction or gravity will carry it to the terminal end of the canal in spite of the law of pneumatics, or that a little agitation with a cotton swab, will do it. But having a pool of solution through which the instrument passes in and out with a portion of the gum adhering to it, the rapid churning process must at length expel all the air, and fill with gum, to completion the entire length of the root.

Do not be afraid of wasting time in this operation. It is time and patience that conquers. Up to this point the rubber dam must be in place to keep the cavity dry. When you feel quite certain that the gum has filled the root, add a drop of water to the cavity. The alcohol, having a greater affinity for the water than for the gum, will leave the gum a firm deposit in the root canal and unite with the water. This process takes a little time also. You will recognize the firm deposit by the increasing difficulty in passing the broach into the canal. Wipe the cavity as dry as possible with bibulous paper, and give a little time for evaporation; then when dry, repeat the operation with the gum, and wiping out all the superfluous gum, leave the cavity for a short time for the evaporation of the alcohol. In the meantime the filling material may be prepared in case it should be advisable to fill at the same sitting. If no unfavorable conditions of the peridental membrane have attended the previous treatment. I have found no unfavorable symptoms follow an immediate filling. If unfavorable indications have attended the operation, I deem it wise to dismiss my patient for a few days or a week, then treat the cavity with a solution of salicylic acid in alcohol before filling.

I am constrained to say, *do not hurry*. It is better to wait for two weeks after the tooth is considered ready for filling, than to hurry the whole operation through in a few days. Success is born of *thorough manipulation* and *time.—Trans. Ill. S. D. Asso.*

